# Electrically assisted cycling for individuals with type 2 diabetes mellitus: a pilot randomized controlled trial

**DOI:** 10.1186/s40814-023-01283-5

**Published:** 2023-04-18

**Authors:** Jessica E. Bourne, Sam Leary, Angie Page, Aidan Searle, Clare England, Dylan Thompson, Robert C. Andrews, Charlie Foster, Ashley R. Cooper

**Affiliations:** 1 Centre for Exercise, Nutrition and Health Sciences, School of Policy Studies, University of Bristol, 8 Priory Road, Bristol, BS8 1TZ UK; 2 NIHR Bristol Biomedical Research Centre, University Hospitals Bristol and Weston NHS Foundation Trust and University of Bristol, Bristol, UK; 3 Department for Health, University of Bath, Bath, BA2 7PB UK; 4 Institute of Biomedical and Clinical Sciences, Medical Research, University of Exeter Medical School, RILD Level 3, Barrack Road, Exeter, EX2 5DW Devon UK

**Keywords:** Type 2 diabetes mellitus, Electrically assisted cycling, E-cycling, Physical activity, Pilot randomized controlled trial

## Abstract

**Background:**

Type 2 diabetes mellitus (T2DM) and its associated complications puts considerable strain on healthcare systems. With the global incidence of T2DM increasing, effective disease management is essential. Physical activity (PA) is a key component of T2DM management; however, rates of PA engagement are low in this population. Developing effective and sustainable interventions that encourage PA is a high priority. Electrically assisted bicycles are becoming increasingly popular and may increase PA in healthy adults. This study aimed to provide evidence of the feasibility of conducting a randomized controlled trial to evaluate the efficacy of an e-cycling intervention to increase PA and improve health in individuals with T2DM.

**Methods:**

A parallel-group two-arm randomized, waitlist-controlled pilot study was conducted. Individuals were randomized to either an e-bike intervention or standard care. The intervention incorporated two one-to-one e-bike skills training and behavioural counselling sessions delivered by a community-based cycling charity, followed by a 12-week e-bike loan with two further sessions with the instructors. Feasibility was assessed via measures related to recruitment, retention and intervention implementation. Post-intervention interviews with instructors and participants explored the acceptability of the study procedures and intervention. Clinical, physiological and behavioural outcomes were collected at baseline and post-intervention to evaluate the intervention’s potential.

**Results:**

Forty participants (*M*_age_ = 57) were randomized, of which 34 were recruited from primary care practices. Thirty-five participants were retained in the trial. The intervention was conducted with high fidelity (> 80% content delivered). E-bike training provided participants with the skills, knowledge and confidence needed to e-bike independently. Instructors reported being more confident delivering the skills training than behavioural counselling, despite acknowledging its importance. The study procedures were found to be acceptable to participants. Between-group differences in change during the intervention were indicative of the interventions potential for improving glucose control, health-related quality of life and cardiorespiratory fitness. Increases in overall device measured moderate-to-vigorous PA behaviour following the intervention were found, and there was evidence that this population self-selected to e-cycle at a moderate intensity.

**Conclusions:**

The study’s recruitment, retention, acceptability and potential efficacy support the development of a definitive trial subject to identified refinements.

**Trial registration:**

ISRCTN, ISRCTN67421464. Registered 17/12/2018.

**Supplementary Information:**

The online version contains supplementary material available at 10.1186/s40814-023-01283-5.

## Key messages

- The feasibility and acceptability of conducting an e-cycling randomized controlled trial among individuals with type 2 diabetes were unknown.
- This study showed that individuals could be recruited and retained for this study and that the data collection procedures were acceptable. In addition, the intervention was acceptable to both participants and instructors.
- Following a series of adaptations proposed as part of this study, a fully powered randomized controlled trial is warranted.

## Background

Type 2 diabetes mellitus (T2DM) is associated with micro- and macrovascular complications [1–6] and can lead to significant reductions in quality of life and the onset of depressive symptoms [7, 8].

Regular physical activity (PA) is valuable in the management of T2DM due to its potential to improve glucose control and other cardiovascular risk factors [9–13]. However, many individuals with T2DM fail to meet the current recommendations of at least 150 min of moderate-to-vigorous physical activity (MVPA) per week [14–16]. While engaging in structured exercise can significantly reduce HbA1c [17], such interventions require a significant amount of contact time and expertise making them unfeasible for large-scale implementation. Furthermore, when left to self-manage PA following an intervention, individuals often return to lower levels of PA [18–20]. As such, there is a need to develop novel interventions that promote high engagement and encourage long-term independent PA behaviour while minimizing impact on resources.

Active travel is a means through which to integrate PA into everyday life and improve health [21–23] and is associated with lower BMI [24] in people with T2DM. However, rates of active travel in the UK, especially cycling, are low among individuals with T2DM. While community-based initiatives can serve to increase cycling behaviour [25–28], it is rarely maintained over time [29, 30]. Furthermore, there are a number of barriers to regular cycling that discourage engagement including physical constraints associated with hilly terrain and poor physical fitness, as well as a lack of time and the distance people have to travel [31]. These barriers may be accentuated in individuals with T2DM due to overall lower levels of PA and fitness.

Electrically assisted bicycles (also known as pedelecs) can help overcome some of the barriers to regular cycling by providing electrical assistance when the rider is pedalling leading to reduced physical exertion compared to conventional cycling. Among individuals with T2DM, a 5-month community-based feasibility study led to a 10% increase in power output, a sign of increased fitness [32], likely to be the result of increased PA. Furthermore, e-cycling was perceived as enjoyable with 14 of the 18 participants purchasing an e-bike at the end of the study. Building on this work, an adequately powered randomized controlled trial (RCT), comparing an e-cycling intervention to a control group, is needed to assess the effectiveness of e-cycling on health and behavioural outcomes among adults with T2DM. However, before this can be done, a pilot RCT is needed to determine the feasibility and acceptability of such a trial and of the e-cycling intervention. This pilot RCT will provide information needed to inform a definitive trial.

As such, the primary aim of this study was to conduct a pilot randomized controlled trial to assess the feasibility and acceptability of conducting an e-cycling intervention and its evaluation among individuals with T2DM. The primary aim was addressed by answering the following research questions (RQs):

1. What are the most effective methods of recruiting individuals with T2DM?
2. Are participants’ willing to be randomized, remain in the study and adhere to the data collection methods and what are the rates of harmful events?
3. Can the intervention be implemented as intended?
4. Are the intervention and study procedures acceptable to participants and instructors?

The secondary aim was to examine changes in outcome measures to determine intervention promise. To address this aim, the following research question was answered: 

(5) What is the potential effect of the intervention on a range of individual clinical, physiological and behavioural outcomes?

Previously identified progression criteria will be used to identify whether a definitive trial is appropriate. The progression criteria are reported in the protocol [33] and include:

1. At least 20% of potentially eligible individuals express an interest in being part of the study.
2. At least 80% of eligible individuals (identified through telephone screening and GP study clearance) are successfully randomized.
3. A study retention rate of ≥ 80%.
4. At least 70% of participants in the intervention group attend at least 60% of the intervention sessions.
5. Process evaluation findings suggest that > 80% of participants report the study methodology to be comprehensible and acceptable.

## Methods

The protocol of this registered trial (ISRCTN#: ISRCTN67421464) is published elsewhere [33] and a brief overview is provided here. The CONSORT extension for randomized pilot and feasibility trials [34] is provided in Additional file 1.

### Study design and procedure

The study, named PEDAL2, was a parallel-group, two-arm, randomized waitlist-controlled pilot study comparing an e-cycling intervention against a standard-care waitlist control in adults with T2DM. The single-centre study was conducted in the city of Bristol, England. The study received ethical approval from the NHS Health Research Authority Southwest/Central Bristol Research Ethics Committee (Ref: 18/SW/0164).

Eligible individuals were stratified based on sex and randomly assigned to one of the two study arms in a 1:1 allocation ratio. Randomization occurred after consent and baseline data collection. Most measures were collected at baseline (time 0 [T0]; March 2019 to June 2019) and immediately following the intervention period (T1; August 2019 to November 2020). PA and travel data were collected in the final week of the intervention and e-cycling behaviour was measured throughout the intervention. Interviews were conducted with participants and instructors at the end of the intervention. The trial aimed to recruit 40 participants as reported in the protocol paper [33].

### Patient involvement

In February 2018, two face-to-face PPI events were conducted with seven individuals with T2DM. Attendees were presented with information on study design, data collection methods and intervention content. The attendees gave feedback on their perceptions of these aspects of the study and the design and intervention content was edited accordingly where possible. In addition, attendees reviewed and provided feedback on intervention material to be distributed to participants.

### Recruitment

Potential participants were identified from primary care practices in the Bristol, North Somerset and South Gloucester Clinical Commissioning group (CCG), diabetes education days and Diabetes Support Network groups. All practices within the CCG were invited to act as a participant identification centre (PIC) for the study. Practices that expressed interest in being a PIC were asked to conduct database searches and send study information to all potentially eligible individuals. Information about the study was also shared at local diabetes education days and support groups. Potential participants had eligibility determined by telephone. If eligible to participate, these individuals were asked to obtain GP clearance to engage in PA, including a maximal fitness assessment, and have their blood pressure taken. All participants with GP clearance were invited for baseline testing where consent was obtained. Recruitment began in November 2018, telephone screening began in March 2019 and ended in May 2019.

### Participants

Eligible individuals had a clinical diagnosis of T2DM and were aged between 30 and 70 years. Individuals were ineligible if they self-reported engagement in ≥ 150 min of MVPA per week [35]; took exogenous insulin; had a myocardial infarction or stroke in the past six months or had evidence of end-stage renal failure or liver disease; had uncontrolled hypertension; had any other contraindications to exercise; were not cleared to engage in PA by their GP and/or were unable to read and communicate in English.

### Intervention condition

The intervention content development has been described in detail elsewhere [33]. Briefly, the final intervention utilised 17 behaviour change techniques (BCTs) to target identified barriers and enablers to e-cycling in the same population. These BCTs were delivered through two one-to-one e-bike training sessions and during two sessions conducted during a 12-week e-bike loan.

The intervention was delivered by a Bristol-based cycling charity, Life Cycle UK. Instructors were fully qualified National Standard cycle instructors, and intervention content training was provided. Training sessions consisted of practical skills training and brief behavioural counselling. Session 1 was mandatory and session 2 was optional based on need and desire as determined by the instructor and participant. Each lasted approximately 120 min. Following the training, participants were provided with an e-bike to take home to use as they desired. Participants were supplied with a pannier, bike lock and maps of cycle routes in the area, as well as details of the Life Cycle UK maintenance service in case of breakdown. Participants were invited to join a social media group (WhatsApp) to share experiences and ride ideas with other participants.

Four weeks after taking the e-bike home participants attended a face-to-face refresher session with the instructor (session 3). This took place at a location of the participant’s choice and lasted approximately 120 min. Session 3 consisted of a practical riding component and brief behavioural counselling. At week eight, the instructor contacted the participants by telephone to discuss their e-cycling activity, barriers that had arisen, potential strategies to overcome these barriers and e-cycling goals for the final four weeks (session 4). At the end of week 12, participants returned the e-bike to Life Cycle UK.

### Control condition

Participants randomly assigned to the waitlist control received two telephone calls at weeks six and ten after baseline testing, to maintain engagement in the study. After post-intervention data collection, participants in the control group were offered e-bike training session 1 and loaned an e-bike for 12 weeks.

### Assessments

#### Feasibility outcomes (RQ 1 and 2)

The following information was recorded: the number of primary care practices approached; the number of practices that agreed to act as a participant identification centre; the number of potentially eligible individuals identified through database searches; the number of individuals attending diabetes education days and Diabetes Support Network groups; and the response rate. Recruitment rates from the three settings, the number of individuals that consented and reasons for not wanting to participate in the study were recorded. Retention rates were determined based on the number of individuals that completed the post-testing measures. The number of individuals that attended each of the data collection sessions and completed the study measures was recorded and the number and types of adverse events resulting from e-cycling documented.

#### Intervention implementation (RQ 3)

Information on intervention dose, fidelity and adaptations were collected [36]. Intervention dose was determined through recording the number of intervention sessions attended by participants and the volume of additional contact between instructors and participants. Intervention delivery fidelity [37] was determined by the degree to which the intervention content was delivered by the instructor (reported as a percentage) as assessed through instructor completed checklists. Information on intervention adaptations were recorded by instructors and reported descriptively.

#### Study and intervention acceptability (RQ 4)

Acceptability of the intervention and data collection methods were explored through semi-structured interviews. Interview questions for participants focused on perceptions and experiences regarding participation in the intervention and data collection processes. Questions for instructors focused on factors that impacted intervention delivery including intervention content, facilities, time, and burden.

#### Outcome measures (RQ 5)

Outcome measures to be assessed in a future trial were measured, and the potential effectiveness of the intervention on these clinical, physiological and behavioural outcomes were explored. Full details of the outcome measures are provided in the protocol [33]. A primary outcome for a definitive trial was identified based on the feasibility of collecting data and the potential for change.

#### Clinical outcomes

Participants’ BMI was calculated. Fasting bloods were collected to measure glucose and insulin levels, HbA1c, lipids and C-reactive protein. Participants completed a 2-h oral glucose tolerance test followed by frequent blood sampling for glucose and insulin. Incremental area under the curve (iAUC) for glucose and insulin levels was calculated using the trapezoid rule. The Matsuda index was used to examine whole body insulin sensitivity [38], while the original insulinogenic index and total insulinogenic index were used to estimate beta cell function. The insulin secretion-sensitivity index-2 (ISSI-2) was used to assess insulin secretion while taking insulin sensitivity into account and is comparative to the disposition index [39]. Higher values for these indices are associated with better insulin sensitivity and/or insulin secretion. Health-related quality of life was assessed using the Short Form Health Survey [40].

#### Physiological outcomes

Cardiorespiratory fitness was determined by measuring maximum oxygen uptake (VO_2max_) using a continuous incremental ramp maximal exercise test on an electronically braked cycle ergometer (Lode Excalibur, The Netherlands). VO_2max_ was defined as the highest 15-breath moving average for VO_2_ (in absolute [l/min] and relative [ml/kg/min] terms) and Wpeak as the highest power achieved. Criteria for achieving VO_2max_ were (i) respiratory exchange ratio > 1.1; (ii) plateau in VO_2_ (defined as a change of less than 0.05L/min between 30-s time sampling intervals); (iii) ≥ 95% of age-predicted HRpeak (220-age); and/or (iv) volitional exhaustion (accepted as > 17 on the Rating of Perceived Exertion scale).^1^ Heart rate (HR) was monitored using a Polar chest strap integrated with the metabolic cart and cycle ergometer software (Lode Exercise Manager). HRpeak was recorded as the highest values attained in the test. Twenty minutes after completing the incremental fitness assessment participants completed a supramaximal assessment following guidelines outlined by Schaun [43]. The verification assessment was used to enhance the precision of the incremental VO_2max_ rather than provide validation of the incremental VO_2max_ result. Therefore, the highest of the two tests was used.

Whole body fat and regional fat and lean mass were assessed using dual-energy X-ray absorptiometry (Hologic Discovery W, QDR software version 12.4.2, Bedford, MA). Images were analysed using the manufacturer’s software. Peripheral quantitative computer tomography (pQCT; XCT 3000 scanner; Stratec, Medizintechnik GmbH, Pforzheim, Germany) was used to assess intermuscular adipose tissue, muscle density, muscle cross-section area (mCSA) and subcutaneous fat area at 33% of the femur length proximally to the lateral femoral epicondyle based on bone length measured. pQCT images were analysed using ImageJ [44] and the method proposed by Owen and colleagues [45]. Images were analysed at the end of the trial. 

#### Behavioural outcomes

##### Hip-worn accelerometer measured physical activity

Time spent in MVPA at baseline and in the final week of the intervention was measured over seven consecutive days using the ActiGraph accelerometer worn on the hip (GT3X; Pensacola, USA). The accelerometer was worn during waking hours and removed for bathing or swimming. Raw monitor data were analysed using the manufacturer’s software (ActiLife v6.13.4; ActiGraph, Pensacola, FL, USA). Data collection protocols and processing are described in Additional file 2.

##### PA intensity due to e-cycling

PA attributable to e-cycling was determined using GPS (Qstarz International Co. Ltd., Taiwan), travel diary data and integrated HR and accelerometer data (Actiheart, CamNtech, Cambridge, UK) worn in the final week of the e-cycling intervention. Specifically, GPS and travel diary-derived temporal trip data for each mode of transport was matched with Actiheart data in Python 3, v.3.7.5. Since the waist-worn Actigraph poorly records PA when cycling [46], Actiheart data were collected specifically to address this outcome. The group calibrated branched equation model [47] was used to calculate instantaneous PA energy expenditure (kcal/kg/min). Sleeping HR was averaged across wear days and entered. Resting energy expenditure was estimated using the Schofield Eqs. [48] and was used in the estimation of metabolic equivalents (METs). The amount of time spent in MVPA (≥ 3 METs) associated with e-cycling was reported in relation to the total amount of time spent e-cycling and total MET minutes from e-cycling were reported. Mean HR during e-cycling was determined and expressed as percentage of HR maximum as determined from the maximal fitness test.

##### E-cycling journeys during the intervention

The total distance travelled on the e-bike was measured using the e-bike odometers. Frequency, duration and distance of e-bike journeys were determined using a GPS bicycle computer (Garmin Edge 130) attached to the e-bike and/or the paper activity logbook. Average weekly distance and duration was determined by dividing the total distance recorded by the number of weeks the e-bike was on loan.

### Analyses

#### Quantitative analyses

Baseline characteristics were summarised by condition using descriptive statistics. Feasibility and implementation outcomes were expressed as frequencies and percentages, with confidence intervals provided when reporting outcomes pertaining to the progression criteria [33]. Any adverse events were described. Evidence of intervention promise (i.e. whether the intervention can lead to positive changes in outcome measures) were examined using comparison of change scores between conditions for all secondary outcome measures (except e-cycling during the intervention). The difference in change scores between conditions is presented with 95% confidence intervals, and *p* values were not considered as the study was not powered to detect effectiveness. Participants were included in the analysis if they provided data at both baseline and post-intervention. Analyses were carried out using Excel and Stata 16 statistical software.

#### Qualitative analyses

Interview data were analysed using the framework method [49] and guided by Gale and colleagues seven-stage analysis process [50] (Additional file 3).

## Results

### Trial feasibility (RQ 1 and 2)

#### Recruitment, randomization and retention

Of 52 regional primary care practices, 31 expressed interest in participation of which 20 were selected to encompass a range of socio-economic areas. Of these 20, 12 completed the searches and sent out study information. Eight practices did not engage in communication from the researchers following the original expression of interest. Of 1855 individuals sent study information, 85 (4.6%) expressed an interest in participating in the study. Study information was shared at three Diabetes Support Network meetings, with two of 41 individuals requesting further information (4.9%), and at six diabetes education days of which 12/150 individuals expressed interest in participating (8.0%). With an estimated 50,255 individuals, of all ages, living with T2DM in the BNSSG CCG in 2019 (National Diabetes Association) approximately 4.1% (*n* = 2046) of potentially eligible individuals received information about the study (95%CI 3.9, 5.9%).

**Fig. 1:**
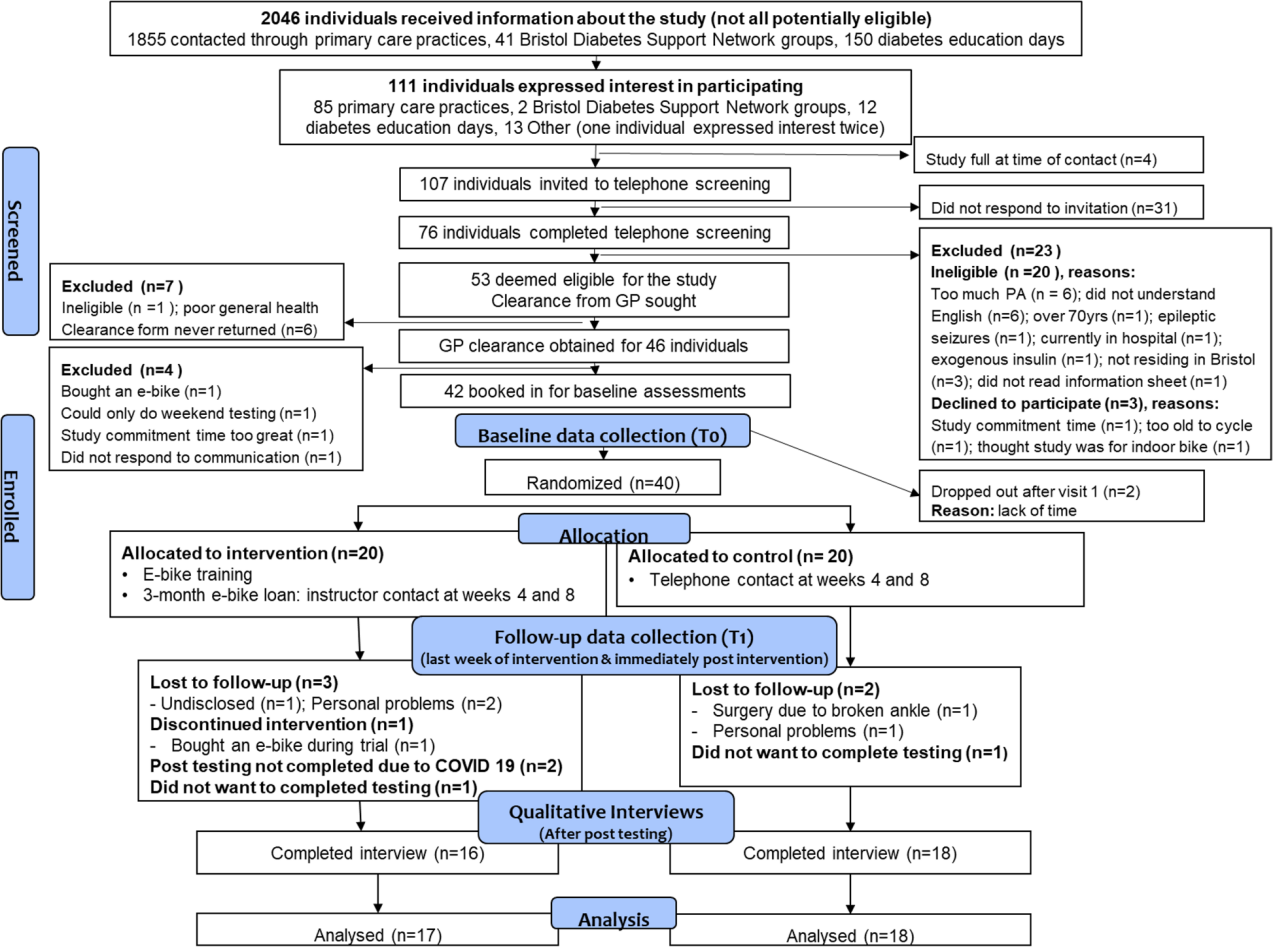
Flow diagram of participants through the trial

In total, 111 individuals expressed an interest in the study, of whom 53 were eligible to participate based on telephone screening (Fig. 1). GP clearance to participate was obtained from 46 of these individuals and 42 consented to participate. Two participants dropped out after visit one; consequently, 40 individuals (95.2%) completed baseline assessments and were randomized, representing 87.0% of participants identified as eligible for the study (95%CI 73.7, 95.1%). Baseline demographic characteristics of the forty participants randomized to the study are displayed in Table 1.

**Table 1:** Baseline demographic characteristics of all study participants, by condition

Variable	Intervention (*n* = 20)	Control (*n* = 20)
Age (year), mean (SD)	57.9 (8.9)	56.2 (8.4)
Gender (*n*, % female)	8 (40)	7 (35)
Ethnicity (*n*, % white)	18 (90)	12 (60)
Employment status (*n*, % working full or part time)	13 (65)	13(65)
Household income (*n*, %)^a^		
£24,999	8 (40)	6 (31.6)
£25–£74,999	9 (45) 11(55)	6 (31.6) 11(57.9)
£75,000 +	1 (5)	2 (10.5)
Body mass index, kg/m^2^, mean (SD)	33.93 (7.18)	32.23 (5.65)
HbA1c, mmol/mol, mean (SD)	54.05 (10.71)	59.22 (20.90)^b^

^a^ One individual did not report household income in the control condition. ^b^*n* = 18

Seventy-six individuals provided reasons for not wanting to participate. A full least of the reasons provided for not wanting to participate are provided in Additional file 4. Based on the eligibility criteria, 41 of these individuals (53.5%) would not have been eligible for the study.

The study had a retention rate of 87.5% (95%CI 73.2, 95.8%, *n* = 35). Reasons for discontinuing the study are provided in Fig. 1.

### Attendance at data collection sessions

All 40 participants attended baseline testing. Three participants did not want to participate in face-to-face post-intervention testing but completed the telephone interview. One of these participants discontinued the intervention due to purchasing an e-bike. Two participants did not complete face-to-face post-intervention testing due to COVID-19 but completed the interview (Fig. 1).

### Collection of clinical, behavioural and physiological outcomes

Completion rates of fasting blood sampling were high (97.5 and 70% for baseline and post-testing respectively). A total of 92.5% of participants completed the OGTT and frequent blood sampling at baseline and 62.5% at post-testing. The primary reason for not collecting blood was a failure to obtain blood from the participant by the clinical staff.

Overall, 87.5 and 60.0% of participants completed the incremental fitness assessment and provided valid data at baseline and post-testing, respectively. Of those that completed the incremental fitness assessment, a further three, at baseline and post-testing, did not complete the supramaximal verification assessment.

High rates of completion, with valid data, were obtained for the body composition scans, leg scans, GPS and Actigraph accelerometer monitoring and the travel diary. Due to negative skin reactions, lower rates of adherence were reported for the Actiheart physical activity monitor. Eighty-five percent of participants completed the interviews at post-testing.

Of the 16 participants that completed the e-cycling intervention, odometer data were available for 14. Trip data collected from the GPS and/or logbook were available for 13 of the 16 participants (81.3%).

### Harmful incidents reported while e-cycling

Six harmful events were reported from three participants (three during e-bike training, three during the loan period), with the majority being low-speed falls resulting in minor or no injury. One participant required hospitalisation due to a broken limb following a low-speed fall. Specifically, the participant lost balance and the bike tipped over while trying to turn at low speed, this resulted in a broken left elbow. After three weeks, the participant was cleared by the GP to continue with the cycle training.

### Intervention implementation (RQ 3)

#### Intervention dose

Prior to beginning the intervention one participant withdrew for reasons unrelated to the study. Four participants took the e-bike home after session 1 and 12 after session 2. Three participants (16.0%) required additional training, as determined by the instructors, prior to taking the e-bike home. The median number of sessions prior to taking the e-bike home was two (Table 2). Prior to session 3, two participants withdrew from the study. Of the 17 that remained, 10 (59.0%) attended session 3. Two participants had additional face-to-face training due to lack of riding confidence. One participant returned their e-bike prior to session 4 due to purchasing an e-bike and choosing not to participate in the intervention. Of the 16 individuals remaining in the intervention, six (37.5%) completed session 4. Overall, 80% of participants (*n* = 20) attended at least half of the intervention sessions (including those that were mandatory). The median e-bike loan period for the 16 participants that remained in the intervention was 14 (IQR 13, 17) weeks. The median time spent in contact with the instructor was 240 (IQR 172.5, 367.5) min.

**Table 2:** Number of e-bike sessions attended and average duration

	E-bike training phase			E-bike loan phase		
Session	Session 1 (*n* = 19)^a^	Session 2 (*n* = 19)^a^	Additional sessions	Session 3 (*n* = 17)^a^	Additional sessions	Session 4 (*n* = 16)^a^
n	19	15^b^	3^c^	10	2^c^	6
%	100	79	16	59	12	37.5
Median duration (IQR), minutes	120 (120,120)	120 (105,120)	150 (135,275)	120 (97.5,120)	135 (127.5, 142.5)	30 (30,52.5)
Notes			2 participants = 1 session 1 participant = 4 sessions		2 participants = 1 session	

*IQR* interquartile range

^a^*n* represents the number of participants enrolled in the intervention at that time. ^b^Four participants took the e-bike home after session 1 and therefore did not complete session 2. ^c^The number of individuals that had additional e-cycling sessions

Attending the refresher session (session 3) during the e-bike loan period appeared to be associated with greater distance travelled over the course of the intervention, with a median of 153.1 (IQR 139.7, 318.8) km for those that attended session 3 and 49.0 (IQR 20.0, 120.0) km for those that did not attend session 3.

#### Intervention fidelity

Additional file 5 displays the percentage of participants who received each of the components of the four sessions as reported by instructor checklists. All participants completed the National Standards for cycle training level 1 and 90% completed at least eight of the 14 skills from level 2. Behavioural counselling during e-bike training was conducted with high fidelity, with over 80.0% of participants receiving all specified content, with the exception of action planning in session 1 which was largely completed in session 2. Sessions conducted during the e-bike loan phase had high fidelity but were infrequently delivered by instructors (Table 2).

#### Intervention adaptations

Intervention adaptations are reported in Additional file 6. The majority of adaptations related to the omission of behavioural counselling components in a small number of participants (29 adaptations across seven participants).

## Intervention acceptability (RQ 4)

### Participants

#### Study procedures

Participants primarily signed up to the study to trial an e-bike and to gain insight into the impact of e-cycling on their health. As such, being assigned to the control group was disappointing. However, knowing they would have access to the bike at the end of the study reduced this disappointment (Table 3, a). The knowledge and friendly manner of the research staff made participants feel at ease and enjoy their data collection visits (Table 3, b).

**Table 3:** Quotes pertaining to intervention acceptability (RQ4)

Participant quotes	
a	‘It didn’t matter because it was going to be something that would eventually happen. In one way I was looking forward to trialling the bike but it’s something that’s going to happen in the future’ (Control, Male)
b	‘I never thought I would enjoy bloodwork so much as I did with the staff. They were great’ (Control, Female)
c	‘I think for the charts, there were some days where I did multiple journeys, and was really here, there and everywhere. There wasn’t enough space’ (Control, Female)
d	‘The exercise tolerance one, I took trying to get to the maximum seriously. So that was, on both occasions, quite an effort’ (Control, Male)
e	‘I never felt comfortable with the bike that I was given because I found the frame too high’ (Intervention, Male)
f	‘That was really useful actually because I was a bit, you know, “I don’t need to do this,” kind of, thing “I’ve always cycled, I don’t need to be shown what to do.” But it was actually quite useful just to do some basics’ (Intervention, Male)
g	‘Yes, it was pretty good, it reinforced the road awareness that I think is quite important, particularly if you haven't ridden a bike for a while. I feel that was very good at pointing out what you should do at junctions and double checking you're aware of everything and making sure you were looking both ways’ (Intervention, Male)
h	‘[the instructor] was excellent. They did it in stages. You progressed out into the little space they’ve got round the Centre. Once you were fully competent you went out and ventured more to where the bus route is. Then in the end we went round XX and I don’t think I would have ever cycled around XX without [the instructor]’ (Intervention, Female)
i	‘Tell me about the training you received? ‘Which I didn’t get….. I jumped on the bike, rode up and down 50 yards each way “Right, that’s fine, thank you very much” I mean, I wasn't there much more than about 40 min, then I took the bike home, which took me by surprise’ (Intervention, Male)
j	‘We did the 45 min on road and cycle paths. Yes, it was good. Not really necessary, but it was good to do. It was silly little things. When I was following him, we went down a one-way system the wrong way, but it was perfectly fine because the roads are actually saying “bikes allowed” on it. It was just little technicalities, you’re thinking, “Well, is that right or not?” but obviously it was. It’s just to reassure yourself and I asked a few little questions, so it was just putting my mind at rest really. (Intervention, Male)
k	‘I’d lock it up, I’d be less worried about if it got stolen, because it’s, like, mine, so I could do something about it, you know? I haven’t got to explain to someone else how I managed to lose a bike’ (Male, Intervention)
Instructor quotes	
l	‘I think it was good to vocalise them [barriers to e-cycling] and discuss them. I felt people felt that having that discussion was useful. It made them think about the barriers. It made them feel that their concerns were being listened to. So we weren’t just going, “Here’s a bike, get on with it.” (Instructor 02)
m	‘Obviously there is a wide range of skills and confidence levels of the people participating, but it felt like it was set up in such a way that you can quickly breeze through all the skills stuff for people who are relatively component’ (Instructor 03)
n	‘I’d say that not everything was applicable to everyone. It seemed like there were some redundant sections that still needed to be filled in even though it wasn’t relevant for that person’ (Instructor 01)
o	‘We kind of all just were like ships in the night, passing each other at different times. Or, if we did see each other, we were working, so we didn’t get much chance to sit and discuss stuff. I think if you were doing it again it would be good to have a pool of instructors and get them together at the beginning, to have a little chat, and then get them together after the first batch of people has gone through the programme to discuss stuff, as well’ (Instructor 02)

Regarding assessments, acceptability of the GPS to track e-cycling varied. Some participant’s greatly enjoying monitoring their behaviour using the GPS while others found it unmanageable. The travel diary was perceived as a burden, with individuals struggling to recall their journeys or disliking with the diary layout (Table 3, c). Nearly all participants reported having a reaction to the electrodes of the Actiheart monitor including a rash, itching or blistering leading to low adherence to the measure. Participants had no concerns completing the body composition scans or wearing the Actigraph and Qstarz GPS. Some participants reported a general dislike of having blood taken and found cannulation uncomfortable, while others had no concerns about having blood taken. While the maximal exertion test was reported as being difficult participants’ understood that this was the purpose of the assessment (Table 3, d). Despite some suggested changes to specific measures, all participants interviewed found the study design to be acceptable.

#### E-bike intervention

Some participants felt the e-bike they were loaned was too large leading to feelings of discomfort (Table 3, e). Furthermore, the weight of the e-bikes made it difficult to manoeuvre for some participants. These concerns were reported by men and women and echoed by instructors.

Despite an initial belief that the e-bike training would be unnecessary, participants with lots of cycling experience reported learning new skills, particularly concerning how to ride in traffic (Table 3, f). For participants with limited cycling experience, who completed all, or more allocated sessions, the training was perceived as appropriate, in relation to both time and content (Table 3, g). However, for participants with no previous cycling experience, the training was good, but they did not feel ready to ride on the road.

Differences in the perception of the training were dependent on the instructor. Some instructors were perceived as engaged and adapted the lesson to meet the participants’ needs while others felt instructors rushed the training (Table 3, h/i). For participants with lots of cycling experience, a lack of instructor engagement did not impact their confidence, but for those with minimal experience more training was required to increase confidence. Participants who felt the instructors were disengaged reported no follow-up contact.

Completion of a face-to-face follow-up session during the e-bike loan was reported as being enjoyable and educational (Table 3, j). A total of ten participants joined the WhatsApp group. However, dialogue mainly occurred between two participants and was seemingly infrequent with only three-four messages sent each month.

Several participants were worried about the e-bike being stolen and felt they had to plan their journeys around having a safe place to lock the e-bike at the destination. The fear of theft was exacerbated as the e-bike was loaned, and participants were unsure of the implications of e-bike theft. This impacted how some individuals used the bike, with several choosing to ride the e-bike only for leisure where they began and ended their ride at home.

#### Instructors

Three of the four instructors took part in telephone interviews at the end of the study. Instructors reported that the skills training was easy to deliver as it followed the National Standards for bicycle training with which they were familiar. The behavioural counselling was perceived as beneficial to the programme and helped make participants feel supported (Table 3, l). However, the level of comfort initiating and engaging in these conversations varied between instructors and was dependent on their perceived expertise to speak to the intervention content and their ability to encourage positive behaviour change. Instructors who felt confident in their knowledge and ability to have these discussions found them enjoyable.

Due to a wide range of cycling skills among participants the intervention was perceived as needing adaption. Some instructors felt the intervention enabled flexibility, while others felt it was highly prescriptive (Table 3, m/n). Instructors would have liked more training on the intervention content and how adaptations could be made prior to delivery. Furthermore, instructors felt that more sharing of experiences with each other would have been useful early on in the implementation process due to unique nature of this population in comparison to their regular cycle training clients (Table 3, o).

### Clinical, physiological and behavioural outcomes (RQ 5)

#### Clinical outcomes

Participants in the intervention arm saw a decrease in weight, BMI and waist circumference (Table 4). After removing individuals who had changes in their diabetes medication, there was a greater decrease in HbA1c in the intervention group compared to the control group (1.33 mmol/mol vs 0.09 mmol/mol). In the intervention group, there was a decrease in glucose incremental area under curve during the OGTT and a reduction in insulin resistance. Beta cell function did not change following the intervention.

**Table 4:** Mean values in clinical outcomes assessed at baseline and follow-up assessments for intervention and control groups and a comparison of the differences in the change between groups

Outcome	Intervention				Control				Difference in change (CI)
		Pre	Post	Change		Pre	Post	Change	
	*N*	Mean (CI) (^a^Median; IQR)	Mean (CI) (^a^Median; IQR)	Mean (CI) (^a^Median; IQR)	*N*	Mean (CI) (^a^Median; IQR)	Mean (CI) (^a^Median; IQR)	Mean (CI) (^a^Median; IQR)	
Anthropometrics									
Weight, kg	13	95.96 (84.16, 107.76)	94.11 (82.98, 105.23)	− 1.85 (− 4.10, 0.40)	17^b^	97.99 (85.39, 110.59)	97.61 (85.12, 110.11)	− 0.38 (− 1.82, 1.07)	1.47 (− 0.97, 3.91)
BMI, kg/m^2^	13	32.98 (28.64, 37.31)	32.37 (28.15, 36.60)	− 0.60 (− 1.32, 0.11)	17^b^	32.32 (29.21, 35.44)	32.18 (29.13, 35.24)	− 0.14 (− 0.61, 0.33)	0.46 (− 0.32, 1.24)
Waist circumference, cm	13	113.27 (103.75, 122.78)	107.88 98.93, 116.84)	− 5.38 (− 9.09, − 1.68)	17^b^	112.09 (103.43, 120.75)	111.46 (101.93, 120.99)	− 0.63 (− 3.36, 2.10)	4.76 (0.47, 9.04)
Fasting bloods									
HbA1c, mmol/mol	12^c^	55.00 (47.26, 62.74)	53.67 (46.81, 60.52)	− 1.33 (− 3.35, 0.68)	11^c^	52.64 (44.26, 61.01)	52.55 (43.45, 61.45)	− 0.09 (− 1.42, 1.23)	1.24 (− 1.07, 3.56)
Fasting glucose, mmol/L	13	7.84 (6.51, 9.16)	7.62 (6.44, 8.79)	− 0.22 (− 0.70, 0.26)	14^d^	7.14 (5.99, 8.29)	7.59 (5.57, 9.62)	0.45 (− 0.94, 1.84)	0.67 (− 0.77, 2.11)
Fasting insulin, mIU/L	13	20.31 (11.26, 29.35)	18.19 (12.30, 24.09)	− 2.12 (− 7.91, 3.68)	13^d^	16.97 (11.92, 22.02)	18.69 (12.17, 24.22)	1.72 (− 2.48, 5.93)	3.84 (− 2.94, 10.62)
HOMA-B, %	13	94.68 (52.01, 137.36)	93.95 (56.55, 131.36)	− 0.73 (− 16.91, 15.45)	13^d^	92.75 (63.50, 122.01)	105.18 (68.12, 142.24)	12.42 (− 4.41, 29.26)	13.15 (− 8.97, 35.27)
HOMA-IR, unitless	13	2.82 (1.66, 3.98)	2.53 (1.78, 3.29)	− 0.28 (− 1.04, 0.47)	13^d^	2.39 (1.68, 3.11)	2.69 (1.80, 3.59)	0.30 (− 0.30, 0.90)	0.58 (− 0.33, 1.50)
C-reactive protein, mg/L	13	1.0 (0.9, 4)	2.0 (0.9, 4)	0.1 (0, 1.1)	14^d^	2.5 (2, 4)	3.0 (2, 4)	0.0 (0, 1)	
Total cholesterol, mmol/L	13	3.91 (3.30, 4.51)	3.95 (3.38, 4.51)	0.04 (− 0.27, 0.35)	15	4.12 (3.70, 4.54)	4.46 (3.80, 5.12)	0.34 (− 0.14, 0.82)	0.30 (− 0.27. 0.87)
Triglycerides, mmol/L	13	1.82 (1.23, 2.42)	1.81 (1.30, 2.32)	− 0.02 (− 0.26, 0.23)	15	1.57 (1.16, 1.99)	1.51 (1.15, 1.87)	− 0.06 (− 0.26, 0.14)	− 0.04 (− 0.35, 0.26)
HDL cholesterol, mmol/L	13	1.32 (1.02, 1.61)	1.23 (1.01, 1.45)	− 0.08 (− 0.19, 0.02)	15	1.13 (0.98, 1.29)	1.19 (1.02, 1.35)	0.05 (− 0.02, 0.13)	0.14 (0.02, 0.26)
LDL cholesterol, mmol/L	12	1.75 (1.35, 2.15)	1.93 (1.49, 2.36)	0.18 (− 0.88, 0.44)	15	2.26 (1.88, 2.64)	2.58 (1.97, 3.19)	0.32 (− 0.10, 0.74)	0.15 (− 0.36, 0.65)
Non-HDL cholesterol, mmol/L	13	2.59 (2.08, 3.10)	2.72 (2.24, 3.19)	0.12 (− 0.19, 0.44)	15	2.99 (2.63, 3.34)	3.27 (2.66, 3.88)	0.29 (− 0.18, 0.76)	0.16 (− 0.40, 0.72)
Dynamic measures of glucose homeostasis									
iAUC glucose, mmol/L/120 min	9	693.33 (536.90, 849.77)	675.5 (552.48, 798.52)	− 17.83 (− 195.75, 298.5)	10^d^	502.35 (340.81, 663.89)	504.98 (418.29, 591.66)	2.63 (− 89.34, 94.59)	20.46 (− 114.21, 155.13)
iAUC insulin, mIU/L/120 min	9	6515.08 (2823.02, 10,207.14)	6844.58 (3184.05, 10,505.12)	329.5 (− 2685, 3122.25)	10^d^	4744.95 (1674.78, 7815.12)	5201.78 (2134.91, 8268.64)	456.83 (− 412.58, 1326.23)	280.37 (− 1003.66, 1564.40)
Matsuda index, unitless	9	2.29 (0.74, 4.06)	2.43 (1.05, 4.81)	0.13 (− 1.69, 1.15)	10^d^	3.00 (0.91, 6.35)	2.37 (0.64, 4.42)	− 0.64 (− 3.38, 1.29)	− 0.77 (− 1.84, 0.30)
Original insulinogenic index (0–30 min), pmol/mmol^e,f^	10	46.33 (5.39, 77.5)	48.11 (6.23, 97.69)	1.77 (− 28.67, 30.29)	11^d^	59.68 (6, 231)	61.71 (9.6, 148.8)	2.02 (− 178.81, 137.29)	0.24 (− 51.50, 51.99)
Total insulinogenic index (0–120 min), pmol/mmol^f^	9	63.60 (4.17, 135.09)	68.57 (5.60, 200.03)	4.97 (− 25.21, 64.94)	10^d^	63.73 (18.59, 146.83)	61.83 (23.44, 168.59)	− 1.89 (− 43.19, 21.76)	− 6.86 (− 29.96, 16.23)
Insulin secretion-sensitivity index 2, unitless	9	58.57 (13.21, 154.59)	66.74 (18.48, 142.07)	8.17 (− 44.08, 68.90)	10^d^	80.39 (20.82, 199.58)	69.60 (12.15, 121.72)	− 10.79 (− 77.86, 44.34)	− 18.97 (− 50.41, 12.48)
Health-related quality of life									
Physical component	13	64.62 (51.82, 77.41)	68.89 (56.41, 81.38)	4.28 (− 6.79, 15.35)	17	71.54 (62.34, 80.74)	69.67 (58.15, 81.18)	− 1.88 (− 13.05, 9.30)	− 6.15 (− 21.16, 8.85)
Mental component	13	67.42 (56.07, 78.77)	71.02 (60.62, 81.42)	3.60 (− 6.20, 13.40)	17	76.27 (67.95, 84.60)	75.46 (66.73, 84.20)	− 0.81 (− 11.54, 9.92)	− 4.41 (− 18.28, 9.46)

*BMI* body mass index, *CI* confidence interval, *HbA1c* glycated haemoglobin, *HDL* high-density lipoprotein: *HOMA-B* homeostasis model assessment of β-cell function, *HOMA-IR* homeostasis model assessment of insulin resistance, *IQR* interquartile range, *LDL* low-density lipoprotein, *iAUC* incremental area under the curve

^a^When data are non-normally distributed, based on visual inspection, median and IQR are reported. ^b^Data from one participant dropped due to having a gastric band during the study. ^c^Individuals removed due to change in medication status. ^d^Data from one participant removed due to taking antibiotics at baseline which impacts measures of glucose and insulin, therefore skewing the data. ^e^Original insulinogenic index required blood samples at 0 and 30 min; therefore, two participants not included in other dynamic glucose measures were included in this calculation. ^f^mIU/L was converted to pmol/L. The conversion factor of 1mIU/L = 6 pmol/L was used

HDL cholesterol decreased by 6.8% in the intervention group and increased by 5.3% in the control group. No changes were seen in total cholesterol, triglycerides, LDL cholesterol, non-HDL cholesterol or C-reactive protein following the intervention.

Regarding HRQoL, within-group mean change in the intervention group showed a 6.6% and 5.3% increase in physical and mental health respectively, while the control group showed a 2.6 and 1.1% decrease in physical and mental HRQoL respectively.

#### Physiological outcomes

Following the intervention, within-group mean change showed an increase of 8.8 and 10.7% in absolute (L/min) and relative (ml/kg/min) VO_2max_ respectively (Table 5). In the control group, there was a 2.6 and 1.9% increase in absolute and relative VO_2max_ respectively. In the intervention group, there was an increase in participants’ maximum power which was not seen in the control group. No changes were seen in body composition as measured by DEXA or pQCT scans in either group (Table 5).

**Table 5:** Mean values in physiological and behavioural outcomes assessed at baseline and follow-up assessments for intervention and control groups and a comparison of the differences in the change between groups

Outcome	Experimental				Control				Difference in change (CI)
		Pre	Post	Change		Pre	Post	Change	
	*N*	Mean (CI)	Mean (CI)	Mean (CI)	*N*	Mean (CI)	Mean (CI)	Mean (CI)	
Cardiorespiratory fitness									
Absolute VO_2max_, L/min	9^a^	1.82 (1.34, 2.30)	1.98 (1.35, 2.61)	0.16 (− 0.06, 0.41)	14	2.28 (2.03, 2.53)	2.34 (2.00, 2.67)	0.06 (− 0.11, 0.23)	− 0.10 (− 0.39, 0.18)
Relative VO_2max,_ L/kg/min	9^a^	19.52 (15.30, 23.75)	21.61 (16.17, 27.04)	2.08 (− 0.86, 5.02)	14	23.07 (20.67, 25.46)	23.50 (21.43, 25.57)	0.43 (− 1.13, 2.00)	− 1.65 (− 4.82, 1.51)
Maximum power output, Watts	9^a^	147.99 (110.14, 185.84)	163.91 (112.70, 215.13)	15.92 (− 5.15, 36.99)	14	188.68 (172.95, 204.41)	181.96 (164.91, 199.01)	− 6.72 (− 13.34, − 0.11)	− 22.65 (− 44.17, − 1.12)
Body composition									
Body fat, %	13	34.03 (28.28, 39.78)	33.79 (28.11, 39.48)	− 0.24 (− 0.99, 0.52)	16^b^	33.63 (29.82, 37.42)	33.24 (29.46, 37.02)	− 0.38 (− 1.34, 0.57)	− 0.14 (− 1.35, 1.07)
Fat mass, kg	13	32.4 (24.91, 40.00)	31.28 (24.34, 38.22)	− 1.18 (− 2.65, 0.30)	16^b^	33.73 (24.91, 40.00)	32.96 (26.48, 39.44)	− 0.77 (− 2.20, 0.65)	0.40 (− 1.57, 2.38)
Trunk fat mass, kg	13	17.79 (13.52, 22.06)	17.13 (13.19, 21.07)	− 0.66 (− 1.46, 0.14)	16^b^	19.12 (15.15, 23.06)	18.64 (14.82, 22.47)	− 0.47 (− 1.29, 0.36)	0.20 (− 0.92, 1.31)
Leg fat mass, kg	13	5.03 (3.77, 6.28)	4.86 (3.63, 6.08)	− 0.17 (− 0.41, 0.07)	16^b^	5.10 (3.97, 6.24)	5.02 (3.94, 6.09)	− 0.09 (− 0.31, − 0.14)	0.08 (− 0.23, 0.40)
Lean mass, kg	13	58.78 (50.98, 66.59)	57.54 (49.98, 65.10)	− 1.24 (− 2.18, − 0.31)	16^b^	61.58 (54.53, 68.62)	61.52 (54.33, 68.71)	− 0.05 (− 1.21, 1.10)	1.19 (− 0.28, 2.66)
Leg lean mass, kg	13	10.27 (8.79, 11.75)	10.09 (8.65, 11.54)	− 0.17 (− 0.30, − 0.04)	16^b^	11.00 (9.49, 12.51)	10.97 (9.51, 12.46)	− 0.01 (− 0.21, 0.19)	0.16 (− 0.08, 0.40)
pQCT femur mCSA, cm^2^	12	104.63 (88.50, 120.75)	101.87 (85.94, 117.81)	− 2.76 (− 6.79, 1.28)	14	121.20 (105.66, 136.73)	119.99 (104.85, 135.12)	− 1.01 (− 3.64, 1.62)	1.75 (− 2.64, 6.13)
pQCT femur density, mg/cm^3^	12	61.63 (57.54, 65.71)	61.56 (57.25, 65.87)	− 0.07 (− 1.10, 0.97)	14	61.78 (58.06, 65.50)	62.44 (59.37, 65.52)	− 0.03 (− 1.78, 1.72)	0.04 (− 2.09, 2.17)
pQCT IMAT area, cm^2^	12	19.99 (15.35, 24.63)	19.25 (14.86, 23.65)	− 0.74 (− 1.09, 0.43)	14	23.94 (16.98, 30.90)	22.46 (17.64, 27.28)	− 0.75 (− 3.48, 1.98)	− 0.01 (− 3.20, 3.18)
pQCT SAT area, cm^2^	12	48.97 (30.24, 67.70)	46.18 (27.08, 65.28)	− 2.79 (− 5.47, − 0.12)	14	41.62 (25.94, 57.31)	41.97 (25.50, 58.45)	0.01 (− 2.34, 2.36)	2.8 (− 0.60, 6.20)
Physical activity									
Daily MVPA, minutes	11	49.12 (25.12, 73.11)	51.65 (24.82, 78.48)	2.53 (− 12.10, 17.16)	15	46.72 (33.39, 60.05)	42.77 (26.93, 58.62)	− 3.95 (− 15.85, 7.95)	− 6.91 (− 15.17, 1.35)
Weekly MVPA, minutes	11	343.83 (175.86, 511.79)	361.55 (173.72, 549.37)	17.72 (− 84.72, 120.15)	15	327.06 (233.75, 420.38)	299.42 (188.53, 410.31)	− 27.64 (− 110.94, 55.66)	− 45.36 (− 170.33, 79.61)

*CI* confidence interval, *IMAT* intramuscular adipose tissue, *mCSA* muscle cross-sectional area, *MVPA* moderate to vigorous physical activity, *pQCT* peripheral quantitative computer tomography, *SAT* subcutaneous adipose tissue, *VO*_*2max*_ maximum oxygen uptake

^a^One participant removed have was told to restrain from exercise for 5 weeks during e-bike intervention due to exploratory surgery. ^b^One participant removed as had a gastric band

#### Behavioural outcomes

The average daily wear time was 869.83(± 93.29) min at baseline and 855.28(± 84.04) min at post-testing, while the average number of valid days was 5.40(± 2.21) days at baseline and 5.17(± 2.25) days at post-testing. There was a mean increase of 17.72(95%CI − 84.72, 120.15) min of MVPA per week in the intervention group compared to a decrease of 27.60(95%CI − 110.9, 55.7) min in weekly MVPA in the control group based on hip mounted accelerometry (Table 5).

Of the six participants that wore the Actiheart during e-cycling, four engaged in e-cycling at a moderate-intensity zone based on METs (Table 6). Percentage of HR_max_ showed that five of the six participants were engaging in moderate-intensity activity during e-cycling, based on 65% of HR_max_ indicating a moderate-intensity activity [51].

**Table 6:** Physical activity associated with e-cycling during post-testing

Participant	Total time spent e-cycling, minutes	Mean heart rate while e-cycling, bpm (SD)	Percentage of HR max	Total time spent in MVPA while e-cycling (≤ 3 METs), minutes	Average METs while e-cycling	Total MET minutes from e-cycling
P1	82.25	109.57 (14.87)	66.01	66	3.81 (1.24)	313.12
P2	58	76.78 (3.42)	59.99	6	2.61 (0.48)	151.40
P3	230	113.92 (14.95)	81.96	212.25	5.08 (1.42)	1168.88
P4	53.5	108.06 (14.00)	67.96	34.75	3.54 (1.56)	189.27
P5	439.75	130.56 (11.04)	75.47	429.5	5.28 (1.15)	2320.05
P6	65.75	112.43 (7.85)	83.90	3	2.24 (0.56)	147.50

*MVPA* moderate to vigorous physical activity, *MET* metabolic equivalent of task, *bpm* beats per minute

Of the 14 participants for which odometer data were available, the median distance travelled during the e-bike loan period was 144.20(IQR 66.00, 284.25) km. Men cycled a median of 261.50(IQR 43.75, 418.75) km, while women cycled a median of 139.50(IQR 121.50, 144.30) km. The median number of trips over the loan period was 22(IQR 12, 33). The median weekly distance cycling was 10.36(IQR 3.94, 18.00) km and median duration was 66.00(IQR 32.00, 94.00) min.

## Discussion

The trial demonstrated that it is feasible to recruit, randomize and retain individuals with T2DM into an e-cycling trial and administer and evaluate a range of outcome measures that were largely acceptable to participants. While the study was not sufficiently powered, the potentially favourable effects of the intervention on clinical, physiological and behavioural outcomes warrant further investigation in a full-scale RCT, subject to the procedural amendments and intervention refinement which are outlined in Additional file 6.

### Study feasibility

The diabetes education days reported the highest response rate; however, due to their extensive reach (90.7% of those reached), targeted mail-outs from primary care practices were the most effective recruitment strategy from which 85% of participants were recruited. This targeted method of recruitment has been effective for recruitment in other PA interventions [52, 53] and has been found to produce a more representative sample than untargeted methods [52] and should therefore be used in a future trial. The mail-out method could be coupled with GP or nurse recommendation during routine appointments in an effort to increase patient uptake.

The response rate was considerably lower in the current study (5.8%) than the 28.3% reported by Cooper and colleagues [32], from which progression criteria for this study was based. However, the current study recruited individuals from a real-world setting, while Cooper and colleagues recruited from an existing pool of 99 individuals. In addition, 50% of GP practices were unable to screen on the five exclusion criteria and the Diabetes Support Network meetings included individuals with both T1DM and T2DM of all ages. Of the individuals that declined to participate (*n* = 76), 53.5% were identified as ineligible. As such, the response rate of 5.8% from those reached is a conservative estimate of those that received information and were eligible.

Of those that expressed interest in the study, 41.4% were eligible to participate. This is a higher “success rate” than other lifestyle intervention trials for T2DM [53, 54], potentially due to the stricter criteria used by other studies based on outcomes of interest (i.e. CVD events, reduced body weight). This suggests that the current eligibility criteria are appropriate for a future RCT, though they should be reviewed based on the selected primary outcome measure. Furthermore, 87% of eligible participants were randomized, therefore meeting the second progression criteria.

The retention rate of 87.5% in the current study, which meets the third progression criteria, is similar to the retention rates of a randomized e-cycling trial among overweight adults [55] and a feasibility e-cycling trial among individuals with T2DM [32]. High retention rates in the current trial are likely due to the ability to trial an e-bike, a primary motivator for participating in the study. While the use of waitlist controls in trials has been debated [56, 57], the inability to blind participants in physical activity interventions and their positive impact on retention means they should be used in future e-cycling interventions. Process evaluations should explore the extent to which individuals in the waitlist control changed their health behaviours following randomization. Furthermore, participants’ willingness to complete measures and return for testing was also influenced by interactions with study staff. Providing a welcoming and relaxed environment has been found to be an effective strategy for maintaining participants in clinical research, but one that is often overlooked [58, 59].

While the Actiheart accelerometer has been found be a reliable and valid measure of PA energy expenditure, including cycling, through integration of HR and accelerometry [60], it caused major skin irritations and therefore compliance was low. Other studies have reported the same issue with this device [61–63]. In comparison, the Actigraph accelerometer, while having high compliance and providing a general measure of PA behaviour, poorly identifies cycling [46]. Alternative ways of assessing the intensity of e-cycling should be used that incorporate HR. This could include using the Actiheart with a strap rather than electrodes or using a watch-based device which may increase compliance and reduce burden.

Despite some specific methodological changes suggested, overall all participants interviewed found the study procedures to be acceptable, thereby meeting progression criteria five and suggesting this design is suitable for a definitive trial.

### Harmful incidents

Most harmful events recorded were low-speed falls associated with loss of balance. Some participants loss of balance may have been due to the weight and/or size of the e-bike. E-bike weight was a commonly reported concern for participants and has been reported in previous research, particularly among older adults and women [64]. Both independently, and in combination, aging and T2DM are associated with a reduction in balance and increased risk of falls [65–67]. Furthermore, balance issues have been associated with e-bike incidents [68]. PA can positively impact balance in older adults and those with T2DM [69–71]; therefore, to reduce the likelihood of balance-related incidents, a future trial should provide e-bikes that are slightly smaller in size than would be conventional for the participants’ height to help them manage the weight of the e-bike by being able to firmly place their feet on the ground, helping with stability and control.

### Intervention implementation

The intervention was feasible to deliver by a community-based organisation and their certified instructors. However, tailoring of the intervention was required based on the wide range of cycling skill level in the sample. Given the costs associated with the provision of instruction (£5556 on instruction and staff administration, approximately £78 per session delivered), it may be important to consider the base level of cycling ability of participants. During recruitment, all participants stated they had some degree of cycling experience. However, during training, two participants disclosed having no cycling experience. While it is important to provide an intervention that is accessible to all, individuals should have basic knowledge of how to ride a bike prior to entering a cycling trial, to maximize the chances of intervention success. Individuals that do not know how to ride a bike could be directed to free community “learn to cycle” initiatives such as are offered by LCUK. Potential participants could also be invited to an e-bike taster session as part of recruitment to determine whether they feel e-cycling is appropriate for them.

Despite a high level of tailoring, the degree of adherence to delivering the skills training and behavioural counselling was high. The high adherence to the intervention components may be reflective of the comprehensive resources developed, which were reported to be useful, and the instructor’s previous experience delivering bicycle skills training and engaging with individuals in this manner. However, the study relied on self-report measures, which produce higher ratings of fidelity than observer reports [72, 73]. A future trial should incorporate independent assessments of fidelity such as observation of sessions [37, 74]. While it would not be feasible to observe every session conducted, each instructor should have each session observed at least once. In addition, different domains of fidelity should be examined including assessments of instructor training on the intervention, receipt of the intervention and engagement by the participants [37]. These domains are infrequently examined in the evaluation of PA interventions but are important for intervention scale-up and sustainability [72].

In the current study, 80% of participants (*n* = 20) completed at least half of the intervention sessions including the optional sessions. E-bike training was perceived as appropriate, both in time and length, by the majority of participants, providing them with the skills and confidence needed to cycle independently. As such, it is recommended that a future trial should include at least one mandatory training session and a second optional session based on need and desire. E-bike sessions during the loan period were often deemed unnecessary, by both the participant and instructor and as such were infrequently conducted. Instructors perceived these sessions to be optional in many cases. However, those that attended the face-to-face refresher sessions reported them to be enjoyable and increased the participants feelings of support. Furthermore, attendance at a face-to-face session was associated with greater e-cycling during the trial. Instructors that were perceived as disengagement rarely conducted e-bike loan sessions and this negatively impacted the cycling of less experienced riders. In a future trial, face-to-face e-bike sessions during the loan period should be conducted at least once and a second optional session should be offered, particular to participants who are struggling to ride.

In addition, a future trial should include more comprehensive training prior to intervention delivery to ensure instructors feel confident delivering all aspects of the intervention and promote intervention buy in. Instructors should be advised on the importance of conducting refresher sessions as a key component of the intervention and they should strive to encourage participant attendance. Sersli and colleagues [25] recommend the use of refresher sessions following their review of conventional bicycle training as a means of increasing cycling behaviour. In addition, peer support groups should be conducted to enable instructors to share and learn from the experiences of others, particularly when conducting training in an unfamiliar population.

### E-cycling and health

Improvements in glucose control and insulin sensitivity were reported in the intervention group. These outcomes are important in the management of T2DM given the detrimental effects of hyperglycaemia and glucose variability in this population [75–77]. This is similar to findings reported by Peterman and colleagues [78] and suggests reduced glucotoxicity. Furthermore, the degree of change in HbA1c in the intervention group, following removal of those who changed their medication, is similar to other exercise interventions in the same population [11, 79]. Given this finding, as well as the ease with which fasting bloods were collected, the inclusion of HbA1c in the majority of physical activity interventions for individuals with T2DM and the diagnostic use of this outcome in clinical settings, it is recommended that a definitive trial selects HbA1c as a primary outcome. However, every effort should be made to include dynamic measures of glucose control and insulin sensitivity as secondary outcomes where possible given the strong link with cardiovascular disease and mortality in individuals with T2DM [77, 80, 81].

In the intervention group, there were improvements in mental and physical HRQoL, a clinically important outcome in the management of diabetes [82]. Similar improvements in HRQoL following engagement in e-cycling have been reported in older adults [83] and are supported by qualitative findings highlighting the enjoyment associated with e-cycling [64, 84].

Furthermore, the intervention was associated with increased cardiorespiratory fitness with a within-group mean increase of 2.10 ml/kg/min in the intervention group. While positive, this within-group change is lower than reported in previous exercise trials in individuals with T2DM [79] and less than the commonly cited clinically meaningful change in VO_2max_ of 3.5 ml/kg/min [85]. However, reduced risk of morbidity and mortality has been associated with lower increases in fitness [86, 87] and the degree of change is similar to previous e-cycling research in inactive and T2DM populations [32, 55, 78, 88]. Changes in health outcomes may be due to changes in PA behaviour. The current study reported an 18-min increase in accumulated average weekly MVPA in the intervention group and a 28-min decrease in the control group. Visual inspection of the Actigraph data during e-cycling revealed that, based on traditional approaches, e-cycling was being classified as a sedentary or light activity. As such, the changes in PA reported in the intervention group represent activity above and beyond that associated with e-cycling. Regarding e-cycling behaviour, participants rode a median weekly distance of 10.36 km for a duration of 66 min. This is substantially lower than other e-bike trials with distances of approximately 70 km per week for around 200 min [55, 78]. However, these studies set weekly cycling goals for participants to reach. Studies exploring free-living e-cycling behaviour report lower weekly distances of between 21 and 37 km [32, 88]. Despite lower weekly cycling distance, in the current trial individuals self-selected to e-cycling at a moderate-intensity (Mean%HR_max_ = 72.5 bpm) in line with other e-cycling studies [89]. The average HR associated with e-cycling was lower in the current trial (110.0 ± 17.5 bpm) than reported by Cooper and colleagues in the same clinical population (125.2 ± 18.1 bpm) [32]. Differences in HR could have been due to participants in the current trial having substantially higher BMI and lower cardiorespiratory fitness at baseline than the sample reported by Cooper and colleagues’. These findings demonstrate that individuals with extremely low fitness and high BMI self-select an e-cycling intensity within a moderate-intensity zone that can positively impact health.

### Strengths and limitations

This is the first study to examine the feasibility and acceptability of conducting a pilot RCT examining an e-cycling intervention in adults with T2DM. However, there are some limitations that must be acknowledged. The individuals in this study were highly motivated to understand their own health and make behavioural changes, therefore self-selecting to participate. As such, the findings may not be generalisable to others with T2DM. However, recruiting from primary care practices, in different socio-economic areas, is likely to have led to a more diverse group of people being reached than previous e-cycling trials [32, 55, 90].

The main strength of this study is the use of a longitudinal randomized controlled design to explore the potential impact of e-cycling on a range of outcomes. The detailed information gained from the quantitative and qualitative methods can be used to help guide the selection of outcome measures and to amend the intervention for a future trial. A rigorous approach was taken to qualitative analysis to increase trustworthiness in the current findings. However, a potential influence of the researcher is unavoidable [91, 92]. The use of participant validation ensured that the interpretation of the interview made by the researcher was consistent with the views of the participant. The quantitative methods used to examine the outcomes built on previous research using robust, objective measures where possible. However, a limitation is that the duration of a diabetes was not recorded in the current trial. Diabetes duration may moderate the association between the intervention and health outcomes, particularly metabolic and quality of life outcomes [93, 94]. As such, this information should be collected as part of a definitive trial.

The measures of implementation used in this study report the degree to which intervention components were delivered. They do not measure the extent to which the participants understood the information provided or engaged with the intervention components at appropriate times during the e-bike loan period. A further limitation of the current study is that the feasibility of collecting data to comprehensively cost the intervention and study procedures was not examined. This information is important to conduct a cost-effectiveness analysis in a definitive trial.

## Conclusions

This study examined the main uncertainties ahead of a definitive trial. Following a series of refinements to the study procedures and intervention, a fully powered RCT is feasible and warranted based on the progression criteria stated. Such a trial will address the unanswered question of the effect of e-cycling on clinical, physiological and behavioural outcomes in individuals with T2DM.

## Supplementary Information

**Additional file 1.** CONSORT extension for Pilot and Feasibility Trials Checklists.

**Additional file 2.** Actigraph processing decisions.

**Additional file 3.** Qualitative analysis approach.

**Additional file 4.** Behavioural intervention completion.

**Additional file 5.** Intervention adaptations.

**Additional file 6.** Future recommandations.

## Data Availability

Data are available upon reasonable request to the corresponding author.

## Abbreviations

BMI: Body mass index
HR: Heart rate
MET: Metabolic equivalent
MVPA: Moderate-to-vigorous physical activity
PA: Physical activity
RCT: Randomized control trial
T2DM: Type 2 diabetes mellitus

## Footnotes

1 The criteria for reaching VO2max was altered from the original protocol paper based on the review of criteria. used in other studies in similar populations [41, 42]. Specifically, the respiratory exchange ratio required to. indicate maximum was reduced from 1.15 to 1.10 and participants were required to reach ≥ 95% age-predicted maximum heart rate.
