# Supplementary material for: Electrically assisted cycling for individuals with type 2 diabetes mellitus: a pilot randomized controlled trial
Source: Pilot Feasibility Stud. 2023 Apr 18;9:60. doi: 10.1186/s40814-023-01283-5 (PMC10111297; doi:10.1186/s40814-023-01283-5)
Supplement: Supplementary file 2 — Additional file 2. Actigraph processing decisions. [file 40814_2023_1283_MOESM2_ESM.docx]

| **Additional File 2.** Actigraph accelerometer procedures and settings | | |
| --- | --- | --- |
| Procedure | Settings | Justification |
| Data collection | | |
| Initialising | Initialized to start recording the day after distribution and to collect data for seven days including a weekend | Used in multiple accelerometery studies with the goal of providing at least four days of valid data. |
| Sampling frequency | 30Hz | For this make of accelerometer this is the only option of sampling frequency. |
| Protocol | ActiGraph GT3X monitor worn on the right hip | Hip worn found to be superior for measuring locomotion, compared to wrist (1) |
| Wear time | Waking hours (except when swimming/bathing/showering) | Wearing the accelerometer on the hip during sleeping could be uncomfortable. Therefore, the research team decided to request the accelerometer was only worn for waking hours. This protocol has been used with older adults (2) |
| Data processing criteria | | |
| Valid length of day | ≥ 10 hours (600 minutes in duration) | This length has been recommended as appropriate to capture sufficient data during the day (3) and has consistently been used in other studies with older adults (4, 5) |
| Days required | ≥ 3 days of valid wear time | Hart and colleagues (6) and Dowd and colleagues (7) suggest a minimum of three days of valid data is required. Migueles and colleagues (3) examined the trade-off between amount of data rejected and the study sample size. Given the small sample size in the current study three days of data meant the inclusion of three participants at baseline and five at post testing who would have been excluded if the requirement of four valid days was set |
| Epoch length | 60-seconds | The activity cut-off points developed by Sasaki and colleagues (8) were validated using 60s cut points. Migueles and colleagues (3) argue that it is important to use the same epoch length from which the cut-off activity points were derived as to limit error in activity classification. |
| Zero counts | ≥ 90 minutes of zeros allowing for 2 mins of activity when placed between 2 30min windows (9) | Time spent in sedentary behaviour increases with age (10) which may lead to increased risk of misclassifying sedentary time as non-wear time. The choi et al (9) algorithm for detection of non-wear time has been proposed for use with older adults (3) and has been used by other studies (5). This algorithm was validated using vector magnitude derived from tri-axial accelerometers.  The use of an automated filter method has been found to be as accurate as combining automated filters with activity logbooks (11). |
| Spurious data | >15000cpm | Used by Audrey and colleagues (5) and by other studies(11) |
| Missing data | No imputation – only use those with enough days and wear time | It is not considered appropriate to impute missing data (11). |
| Filter | Normal | Migueles et al (3) recommend using the same filter as was used in the validation study for cut points being employed in the study. Therefore normal frequency filter will be applied to the Sasaki cut points (8). |
| Activity cut-off points | Light ≤ 2690  MVPA≥2691 | Sasaki et al (2011) cut points has been found to be the most appropriate activity cut points for use in adults (3) and will therefore be used in the current study. |
| Axis of analysis | Vector magnitude (VM) | Triaxial accelerometry has been reported to provide a better estimate of PA than uni-axial with some research suggesting this is particularly important when looking at activities such as cycling (11, 12). The Sasaki et al (8) cut points used in this study were developed using the three axis and so this procedure will be followed in the current trial. |

**References**

1. Ellis K, Kerr J, Godbole S, Staudenmayer J, Lanckriet G. Hip and Wrist Accelerometer Algorithms for Free-Living Behavior Classification. Medicine & Science in Sports & Exercise. 2016;48(5):933-40. doi:10.1249/mss.0000000000000840

2. Giné-Garriga M, Sansano-Nadal O, Tully MA, Caserotti P, Coll-Planas L, Rothenbacher D, et al. Accelerometer-Measured Sedentary and Physical Activity Time and Their Correlates in European Older Adults: The SITLESS Study. The Journals of Gerontology: Series A. 2020;75(9):1754-62. doi:10.1093/gerona/glaa016

3. Migueles JH, Cadenas-Sanchez C, Ekelund U, Delisle Nystrom C, Mora-Gonzalez J, Lof M, et al. Accelerometer Data Collection and Processing Criteria to Assess Physical Activity and Other Outcomes: A Systematic Review and Practical Considerations. Sports Medicine 2017;47(9):1821-45. doi:10.1007/s40279-017-0716-0

4. Jung ME, Locke SR, Bourne JE, Beauchamp MR, Lee T, Singer J, et al. Cardiorespiratory fitness and accelerometer-determined physical activity following one year of free-living high-intensity interval training and moderate-intensity continuous training: a randomized trial. International Journal of Behavioral Nutrition and Physical Activity. 2020;17(1):25. doi:10.1186/s12966-020-00933-8

5. Audrey S, Fisher H, Cooper A, Gaunt D, Garfield K, Metcalfe C, et al. Evaluation of an intervention to promote walking during the commute to work: a cluster randomised controlled trial. BMC Public Health. 2019;19(427):1-13. doi:10.1186/s12889-019-6791-4

6. Hart TL, Swartz AM, Cashin SE, Strath SJ. How many days of monitoring predict physical activity and sedentary behaviour in older adults? International Journal of Behavioral Nutrition and Physical Activity. 2011;8(1):62. doi:10.1186/1479-5868-8-62

7. Dowd KP, Szeklicki R, Minetto MA, Murphy MH, Polito A, Ghigo E, et al. A systematic literature review of reviews on techniques for physical activity measurement in adults: a DEDIPAC study. International Journal of Behavioral Nutrition and Physical Activity. 2018;15(15):1-33. doi:10.1186/s12966-017-0636-2

8. Sasaki JE, John D, Freedson PS. Validation and comparison of ActiGraph activity monitors. Journal of Science and Medicine in Sport. 2011;14(5):411-6. doi:10.1016/j.jsams.2011.04.003

9. Choi L, Liu Z, Matthews CE, Buchowski MS. Validation of accelerometer wear and nonwear time classification algorithm. Medicine & Science in Sports & Exercise. 2011;43(2):357-64. doi:10.1249/MSS.0b013e3181ed61a3

10. Cooper AR, Goodman A, Page AS, Sherar LB, Esliger DW, van Sluijs EMF, et al. Objectively measured physical activity and sedentary time in youth: the International children’s accelerometry database (ICAD). International Journal of Behavioral Nutrition and Physical Activity. 2015;12(1):113. doi:10.1186/s12966-015-0274-5

11. Peeters G, van Gellecum Y, Ryde G, Farías NA, Brown WJ. Is the pain of activity log-books worth the gain in precision when distinguishing wear and non-wear time for tri-axial accelerometers? Journal of Science and Medicine in Sport. 2013;16(6):515-9. doi:10.1016/j.jsams.2012.12.002

12. Smith MP, Horsch A, Standl M, Heinrich J, Schulz H. Uni- and triaxial accelerometric signals agree during daily routine, but show differences between sports. Scientific Reports. 2018;8(15055). doi:10.1038/s41598-018-33288-z
