# Supplementary material for: Electrically assisted cycling for individuals with type 2 diabetes mellitus: a pilot randomized controlled trial
Source: Pilot Feasibility Stud. 2023 Apr 18;9:60. doi: 10.1186/s40814-023-01283-5 (PMC10111297; doi:10.1186/s40814-023-01283-5)
Supplement: Supplementary file 3 — Additional file 3. Qualitative analysis approach. [file 40814_2023_1283_MOESM3_ESM.docx]

**Additional File 3.** Analysis of qualitative interview data

| Procedure for analysis | **Application in the current study** |
| --- | --- |
| Stage 1 Transcription | All interviews were conducted by JEB and transcribed by a university approved transcription service, Transcription UK. The transcripts were checked against the original recordings to ensure reliability. |
| Stage 2 Familiarization | JEB became immersed in the data by listening to each audio recording and reading each transcript, making notes about potential codes and data relating to overarching research questions and general thoughts. AS read four participant interview transcripts (10%) and two instructor interview transcripts (20%). JEB selected the specific transcripts to represent diverse experiences and opinions of participating in the study. |
| Stage 3 Coding | To develop a coding frame JEB and AS independently assigned codes to each segment of the data deemed to be potentially relevant to the research questions from the selected transcripts. An inductive approach to coding was taken. N-Vivo 12 software was used to review, organise, and combine the data. |
| Stage 4 Develop an analytical framework | After coding the initial transcripts, the researchers met to discuss coding and an analytical framework was developed. This consisted of a set of codes, each with a brief description. The two researchers independently coded 2 more participants transcripts and 1 instructor transcript, noting any new codes. The researchers met again to discuss the coding and to revise the initial framework to incorporate new or redefined codes.  At this point the conceptual relationship between codes was considered and similar codes were grouped together into categories, while taking into consideration the research objectives. The researchers did not look beyond what was explicitly said by participants, adopting a semantic approach to identifying categories. In this sense, the categories were a method of organising the data, acting to display common patterns within the dataset. An ‘other’ code was included under each category to avoid ignoring data that does not fit. |
| Stage 5 Applying the analytical framework (Indexing) | JEB used this framework to code the remaining transcripts using NVivo software. If a new code was required as it was not covered by the initial framework, this was discussed with AS before adding to the analytical framework. If a new code or category was added, previously coded transcripts were checked for data relevant to the new code. |
| Stage 6 Charting data into the framework matrix | After finalising codes and categories, a framework matrix was developed. N-Vivo was used to create matrices that encapsulate data from each category and code. Following this, each participants data was described and summarized to develop a chart. This was conducted in Excel and consisted of participants in rows with summaries of categories in columns. The matrix contained summaries of the data for each participant with references to specific examples but not the actual raw data. The framework matrix enabled the investigation of data horizontally (case-by-case) and vertically (to investigate themes and sub-themes). AS checked the summaries of the first 4 transcripts to ensure the summaries captured the essence of the data.  Following stage 6, four participants and one instructor were sent a copy of their interview transcript and an interpretation of the data. They were asked to review their transcribed data and comment if they felt the interpretations represented or misrepresented their views. |
| Stage 7 Mapping and Interpreting the data | The significance and implications of the categories, and how they relate to one another was examined to generate broader categories, while considering the research objectives. This was done by JEB and AS collaboratively. The findings are reported narratively in the results and illustrative quotes presented. |
