# Supplementary material for: Electrically assisted cycling for individuals with type 2 diabetes mellitus: a pilot randomized controlled trial
Source: Pilot Feasibility Stud. 2023 Apr 18;9:60. doi: 10.1186/s40814-023-01283-5 (PMC10111297; doi:10.1186/s40814-023-01283-5)
Supplement: Supplementary file 4 — Additional file 4. Behavioural intervention completion. [file 40814_2023_1283_MOESM4_ESM.docx]

| **Additional File 4** Content of the PEDAL2 intervention and number of individuals that received that content | | | |
| --- | --- | --- | --- |
| **Session** | **Intervention content** | **Number of attendees who received this content (%)** | **Comments** |
| **E-bike training phase** | | |  |
| Session 1 (n=19) | National Skills Level 1. Example activities include carry out a simple bike check, stop quickly under control, look all around when riding without loss of control (8 unique skills) | 19 (100%) | Two participants completed this in session 2 |
|  | National Skills Level 2. Example activities include be able to signal intentions to other road users, make a u-turn, demonstrate a basic understanding of the Highway Code (14 unique skills) | 7 (37%) completed all skills  7 (37%) completed 10-13 skills  3 (16%) completed 8-9 skills | Six participants completed some of this training in session 2 |
|  | Instructor to provide participant with feedback on their e-cycling during and after session | 15 (80%) |  |
|  | Instructor to provide positive encouragement to participant throughout the session | 16 (84%) |  |
|  | Importance of tracking behaviour is discussed and how e-cycling felt. Participant is provided with paper logbook and Garmin GPS (if desired) and encouraged to record their e-cycling activity | 17 (89%) |  |
|  | Instructor to provide verbal information on potential health and emotional benefits associated with physical activity and specifically e-cycling. The potential environmental consequences of e-cycling will also be discussed. | 15 (80%) |  |
|  | Instructor to encourage participant to think about when and where they plan to ride the e-bike and whether alone or in a group and make ride plans | 12 (63%) |  |
| Session 2  *Optional (n = 15) | National Skills Level 3. Example activities include demonstrate how to safely pass queuing traffic, demonstration of how to use roundabouts, how to use multi-lane roads. | 4 (27%) completed all skills  2 (13%) completed 12-13 skills  1 (7%) completed 4 skills | 7 participants (53%) worked on level 2 skills during session 2  1 no checklist |
|  | Instructor to provide participant with feedback on their e-cycling during and after session | 13 (87%) | 1 no checklist |
|  | Instructor to congratulate participant on session | 13 (87%) | 1 no checklist |
|  | Participant encouraged to record their e-cycling in the logbook or online | 12 (80%) | 1 no checklist  1 participant still not confident to ride alone |
|  | Instructor to encourage participant to think about when and where they plan to ride the e-bike and what for. | 12 (80%) | 1 no checklist  1 participant still not confident to ride alone |
|  | Provide participant with cycling maps to help them identify routes. Discuss potential cycling routes with participants and plan how they will get to those routes | 12 (80%) | 1 no checklist  1 participant still not confident to ride alone |
|  | Encourage participant to set specific (i.e., SMART) e-cycling goals for the upcoming e-bike loan period | 13 (87%) | 1 no checklist |
|  | Participant encouraged to identify potential barriers to e-cycling and come up with ways to overcome these barriers | 14 (93%) | 1 no checklist |
| When e-bike taken home (n=19) | Participant provided with helmet, panniers and lights for the duration of the e-bike loan period and cycle maps | 18 (95%) | 1 no checklist |
|  | Participant asked if they wanted to connect with other participants via social media (WhatsApp Group) and provided with information on Life Cycle UK social rides. | 18 (95%) | 1 no checklist |
|  | Participant provided with details of bike breakdown and maintenance service which can be utilized throughout the intervention period | 18 (95%) | 1 no checklist |
| **E-bike loan phase** | | |  |
| Session 3  Location of participants choice  (n = 10) | Participant and instructor ride together at a location of the participants choice | 9 (90%) | 1 completed as phone call |
|  | Instructor to provide feedback to the participant on their riding | 10 (100%) |  |
|  | Instructor and participant to review past 4-weeks of e-cycling. Instructor to provide positive encouragement and to encourage participant to focus on past success | 10 (100%) |  |
|  | Review barriers to e-cycling that have arisen in the past 4-weeks and how these were overcome or could be overcome in the future. | 10 (100%) |  |
|  | Review e-cycling goals and encourage participant to amend if necessary | 10 (100%) | 4 participants report not achieving e-bike goals. Two set new goals |
|  | Participant encouraged to plan where and when they want to ride in the future, discuss potential cycling routes | 10 (100%) | 1 participant unlikely to ride alone yet but was discussed |
|  | Provide participants with details of upcoming group rides at LifeCycle UK that the participant could attend | 10 (100%) | 1 participant given information but unlikely to use as not confident to ride e-bike alone |
| Session 4  Telephone (n=6) | Review of e-cycling behaviour over the past month and instructor to provide feedback | 6 (100%) | 1 session conducted as a face-to-face session |
|  | Instructor to focus on successes and provide positive encouragement | 6 (100%) |  |
|  | Review of barriers that have arisen and discussion on how these were overcome/plan ways to overcome these | 6 (100%) |  |
|  | Review e-cycling goals and encourage participant to amend if necessary | 6 (100%) | 1 participant not achieving goal as still not confident on the e-bike therefore barely riding |
|  | Discuss how and where participants plan to ride in the final 4 weeks. Discuss potential cycling routes | 6 (100%) | 1 participant unlikely to ride alone yet but was discussed |
|  | Provide participants with details of upcoming group rides at LifeCycle UK that the participant could attend | 6 (100%) | 1 participant given information but unlikely to use as not confident to ride e-bike alone |
|  | Participants advised to connect with friends and family and to inform them of their goals to build support and have some accountability | 6 (100%) |  |
|  | Remind participants of the importance for rewarding themselves for achieving their goals or making progress towards their goals | 6 (100%) |  |
