# Supplementary material for: Electrically assisted cycling for individuals with type 2 diabetes mellitus: a pilot randomized controlled trial
Source: Pilot Feasibility Stud. 2023 Apr 18;9:60. doi: 10.1186/s40814-023-01283-5 (PMC10111297; doi:10.1186/s40814-023-01283-5)
Supplement: Supplementary file 5 — Additional file 5. Intervention adaptations. [file 40814_2023_1283_MOESM5_ESM.docx]

| **Additional File 5.** Occurrence and rationale for adaptations made to the intervention as intended | | |
| --- | --- | --- |
| **Adaptation** | **# Participants affected** | **Reported reason(s)** |
| E-bike training phase | | |
| E-bike training conducted at participants home | 1 | Participant unable to find time to come to CREATE centre for training |
| E-bike training lasted longer than 4 weeks | 3 | Participant broke elbow and training had to be paused (n=1); participant required special equipment for the e-bike (n=1); participant went on holiday during the training phase (n=1) |
| Additional e-bike training sessions | 3 | Participant lacked confidence riding the e-bike (n=1); participant required assistance with riding different routes and requested instructor assistance (n=1) |
| National skills level 1 and 2 conducted in session 2 |  | Participants were unable to complete all NS1 and NS2 skills in session 1 therefore these were complete during session 2. |
| Session 1, not delivered BCT: *Feedback on behaviour* | 4 | Reason unknown |
| Session 1, not delivered BCT: *Social support (unspecified)* | 3 | Reason unknown |
| Session 1, not delivered BCT *self-monitoring of behaviour* | 2 | Participants not given logbooks, reason why unknown |
| Session 1, not delivered BCT, *information about health, emotional and social and environmental consequences of physical activity and e-cycling* | 4 | Reason unknown |
| Session 1, not delivered BCT, *Action planning* | 7 | Participants were not at a stage where they would be planning their own journeys. Instructors felt it was more appropriate to wait until session 2, immediately prior to the e-bike being taken home. |
| Checklists not completed but session conducted | 1 | Session 2 for one participant was completed but no checklist completed |
| Session 2, BCT not delivered, *feedback on behaviour* | 1 | Reason unknown |
| Session 2, BCT not delivered, *social support (unspecified)* | 1 | Reason unknown |
| Session 2, BCT not delivered, *feedback on behaviour* | 1 | Reason unknown |
| Session 2, BCT not delivered, *self-monitoring of behaviour* | 1 | Reason unknown |
| Session 2, BCTs not delivered, *action planning, adding objects to the environment* | 2 | Reason unknown (n=1); not sufficiently confident to ride e-bike alone (n=1) |
| Session 2, BCT not delivered, *goal setting (behaviour)* | 1 | Reason unknown |
| E-bike loan phase |  |  |
| E-bike loan period lasted longer than 3-months (+1 week to allow for return) | 6 | Loan period extended due to illness meaning participant unable to ride e-bike (n=2; 22 wks, 20wks);  Post testing appointment could not be coordinated for earlier (n=2; 16 wks, 16wks);  Participant unconfident on e-bike so extended by instructor in effort to increase cycling confident (n=1; 31 wks);  Post testing changed and later cancelled due to COVID-19 (n=1; 23 wks) |
| Session 3, BCTs not delivered, *instruction on how to perform behaviour, demonstration of behaviour, behavioural practice/rehearsal* | 1 | Session conducted over the telephone rather than face to face |
| Session 3 not delivered | 3 | Three participants could not find the time to complete session 3. Four participants, reason not provided by instructor |
| Session 4 not delivered | 10 | No reasons were provided by the instructors for session 4 being missed by 10 participants |
