# Supplementary material for: Electrically assisted cycling for individuals with type 2 diabetes mellitus: a pilot randomized controlled trial
Source: Pilot Feasibility Stud. 2023 Apr 18;9:60. doi: 10.1186/s40814-023-01283-5 (PMC10111297; doi:10.1186/s40814-023-01283-5)
Supplement: Supplementary file 6 — Additional file 6. Future recommandations. [file 40814_2023_1283_MOESM6_ESM.docx]

| **Additional file 6.** Adaptations to a future e-cycling trial based on results of the current pilot and feasibility trial | |
| --- | --- |
| Study procedures | Considerations for future trials |
| Recruitment | Researchers should engage with GP practices, ideally through a clinical research network, to act as participant identification sites |
|  | Database searches that encompass most of the eligibility criteria should be developed by GP practices, and guided by the researchers, to accurately estimate reach |
|  | Ensure that participants have some level of cycling experience prior to entering the trial |
|  | Offer e-bike trial days for interested individuals to determine whether they would like to join the trial |
| Retention | Incorporate a waitlist control to maintain engagement |
|  | Ensure participants are provided with individual reports of their data at the end of the trial |
|  | Ensure research and nursing staff are welcoming and willing to engage with participants |
| Study procedures | Reduce the time participants are required to attend procedural visits where possible |
|  | Conduct all data collection in one setting, ideally in a location that is familiar and easily accessible |
| Outcome measures |  |
| *Blood sampling* | If conducted ensure participants are fully hydrated prior to the appointment and utilize experienced nursing staff where possible to increase chances of bleeding |
| *Fitness assessments* | Participants are willing to complete a fitness assessment despite experiencing discomfort. If conducting and the outcome is of primary interest, consider including the supramaximal test to increase confidence in the results of an incremental fitness assessment without repeated the measure on a different day. |
| *Measurement of physical activity* | Consider using an integrated heart rate and accelerometer device for measuring e-cycling. Actigraph, while low burden, is unable to accurately measure e-cycling and the Actiheart causes discomfort and skin reactions |
| *GPS devices* | Had low participant burden and provided accurate data therefore if required is suitable to use in future trials. |
| *Travel diaries* | Redesign the logbook based on participants feedback and provide an online tool to provide real time trip information. Some form of travel diary is useful to capture the context of trips |
| Intervention | Provide instructors with greater training on the intervention content, including ways to adapt the intervention and a focus on behavioural counselling. Ensure the content is understood by conducting role play sessions and assessing knowledge |
|  | Develop a peer support group for instructors to share their experiences of delivering the intervention |
|  | Ensure instructors are reimbursed for the administration time for organising e-bike sessions and equipment |
|  | Continue to record intervention dose using instructors’ records |
|  | Two e-bike training sessions prior to an e-bike loan is appropriate for most participants. However, adapt the sessions as appropriate for the individual. |
|  | Conduct some training session observations by trained individuals to assess intervention delivery fidelity |
|  | Ensure the size of the e-bikes is appropriate for the skill level of the participant. This will likely require offering a smaller than standard bike for the individuals height |
|  | Encourage participants to attend e-bike sessions during the loan period to increase engagement. |
